# Influence of air pollutants on circulating inflammatory cells and microRNA expression in acute myocardial infarction

**DOI:** 10.1038/s41598-022-09383-7

**Published:** 2022-03-30

**Authors:** Alberto Cecconi, Gonzalo Navarrete, Marcos Garcia-Guimaraes, Alberto Vera, Rafael Blanco-Dominguez, Ancor Sanz-Garcia, Marta Lozano-Prieto, Beatriz Lopez-Melgar, Fernando Rivero, Pilar Martin, Francisco Sanchez-Madrid, Hortensia de la Fuente, Luis Jesus Jimenez-Borreguero, Fernando Alfonso

**Affiliations:** 1 Department of Cardiology, Instituto de Investigación Sanitaria Hospital Universitario de La Princesa (IISP), Universidad Autónoma de Madrid, c/Diego de León 62, 28006 Madrid, Spain; 2 Vascular Pathophysiology Area, Centro Nacional de Investigaciones Cardiovasculares (CNIC), Madrid, Spain; 3 Data Analysis Unit, Instituto de Investigación Sanitaria, Hospital Universitario de La Princesa, Madrid, Spain; 4 Department of Immunology, Instituto de Investigación Sanitaria Hospital Universitario de La Princesa (IISP), Universidad Autónoma de Madrid, c/ Diego de León 62, 28006 Madrid, Spain; 5 Centro de Investigación Biomédica en Red Enfermedades Cardiovasculares (CIBERCV), Madrid, Spain

**Keywords:** Myocardial infarction, miRNA in immune cells, Immunology, Environmental sciences

## Abstract

Air pollutants increase the risk and mortality of myocardial infarction (MI). The aim of this study was to assess the inflammatory changes in circulating immune cells and microRNAs in MIs related to short-term exposure to air pollutants. We studied 192 patients with acute coronary syndromes and 57 controls with stable angina. For each patient, air pollution exposure in the 24-h before admission, was collected. All patients underwent systematic circulating inflammatory cell analyses. According to PM_2.5_ exposure, 31 patients were selected for microRNA analyses. STEMI patients exposed to PM_2.5_ showed a reduction of CD4^+^ regulatory T cells. Furthermore, in STEMI patients the exposure to PM_2.5_ was associated with an increase of miR-146a-5p and miR-423-3p. In STEMI and NSTEMI patients PM_2.5_ exposure was associated with an increase of miR-let-7f-5p. STEMI related to PM_2.5_ short-term exposure is associated with changes involving regulatory T cells, miR-146a-5p and miR-423-3p.

## Introduction

Ambient air pollution is a major health risk factor, leading to cardiovascular and respiratory diseases. Worldwide almost 9 million deaths in 2015 were attributable to air pollution. In Europe an annual attributable mortality of 790 000 people (133 deaths per 100 000) has been estimated. Of this excess of mortality, at least 48% is due to cardiovascular diseases as coronary artery disease (CAD) and ischemic stroke^1^. Chronic and acute exposure to air pollutants are both associated with an increased risk of myocardial infarction (MI)^2,3^. Furthermore, exposure to air pollutant increases the risk of MI-related ventricular arrhythmias and mortality^4^. PM_2.5_ exposure is associated with cardiovascular events through a biological pathway that includes higher leucopoietic activity and arterial inflammation^5^. Although elevation of systemic inflammatory biomarkers as IL-6 and C-reactive protein (CRP) have been associated to short-term exposure to air pollutant^6,7^, the precise inflammatory cell pattern associated with the main air pollutants in patients with ischemic heart disease remains undetermined.

Air pollution-induced atherosclerotic plaque destabilization may be related to different mechanisms including inflammation, thrombogenicity and endothelial dysfunction^8–10^. All these pathologic pathways imply different mRNA transcription processes, and these, in turn, are regulated by distinct microRNAs (miRNAs). Several miRNAs have been reported as responsive to air pollution^11^. However, specific miRNA patterns related to MI secondary to air pollution remain unknown.

The present study was designed to address two main objectives: firstly, to assess the circulating inflammatory cell changes associated with short term exposure to air pollutants in patients presenting with acute MI and, secondly, to define the precise miRNA signature of MI related to short-term exposure to PM_2.5_.

## Methods

### Population of the study

Our tertiary University Hospital, localized in the central core of Madrid, covers an area of 350.000 inhabitants and it is part of the Regional Network for acute ST-segment elevation myocardial infarction (STEMI). We prospectively included all consecutive patients admitted to our center between March 2017 and July 2018 with the diagnosis of STEMI and non-STEMI (NSTEMI) undergoing coronary angiography in the acute phase of the disease. For comparative purposes, we included a control group of patients with stable angina who underwent cardiac catheterization in our institution during the same recruitment period. Demographic data and other relevant clinical information were prospectively collected, including cardiovascular risk factors, previous medical history, Killip-Kimball class at presentation, angiographic information, high-sensitive T-troponin and creatine kinase peak. Exclusion criteria were: MI without obstructive coronary artery disease; coronary artery events no related to acute atherosclerotic plaque destabilization (e.g. spontaneous coronary artery dissection, coronary embolism or vasospastic angina); history of chronic inflammatory disease or concomitant treatment with anti-inflammatory drugs; and lack of data about air pollutant exposure. All patients underwent systematic circulating inflammatory cell analysis. To select plasma samples for miRNAs analysis, at the end of the recruitment period, patients were sorted out according to PM2.5 exposure. Upper and lower values were sex- and age-matched and, eventually, a group of 31 patients, representing high and low exposure, were selected (14 STEMI, 9 NSTEMI and 8 stable angina).

### Air pollutant data collection

Madrid benefits from a network of 24 meteorological stations recording air pollutants concentration. For each patient we collected daily air concentrations of PM_10_ (µg/m^3^), PM_2.5_ (µg/m^3^), NO_2_ (µg/m^3^), SO_2_ (µg/m^3^), NO (µg/m^3^), CO (mg/m^3^), and O_3_ (µg/m^3^) from the closest meteorological station to the patient residence. Each parameter was measured every hour and we obtained the previous 24-h average before hospital admission.

### Blood samples

An arterial blood sample was collected in BD Vacutainer tube (BD Plymouth, UK) at time of catheterization, before heparin administration. Blood samples were processed up to 24 h from collection and during this time they were kept at 4 °C. Plasma samples were obtained by centrifugation at 2000*g* at 4 °C, aliquoted and stored at − 80 °C until total RNA extraction. Plasma samples were tested for the presence of hemolysis using the absorbance at 414 nm in a NanoDrop One spectrophotometer (Thermo Scientific).

### Inflammatory cell analysis

Peripheral Blood Leukocytes (PBLs) were isolated from human blood samples using Ficoll-Isopaque (density = 1.121 g/ml) gradient centrifugation. Human PBLs were incubated with fluorochrome-conjugated antibodies (Supplemental Table 1) for flow cytometry analysis. Membrane staining were performed in phosphate-buffered saline (PBS), 0.5% Bovine Serum Albumin (BSA), 1 mM EDTA during 15 min on ice.

For T cell subsets analysis, the rest of PBLs were cultured overnight in plates coated with 3 µg/ml purified anti-CD3 (ΟΚΤ3 clone, Biolegend) in complete RPMI medium (Gibco) before cell staining. For regulatory T cell evaluation, cells were membrane-stained with anti-CD4 and anti-CD25 and then nuclear staining was performed using the Foxp3 staining buffer set (Miltenyi Biotec), according to the provider’s instructions.

For cytokine production assessment, cells were re-stimulated with 50 ng/ml phorbol myristate acetate (PMA, Sigma Aldrich), 1 µg/ml ionomycin (Sigma Aldrich) and 1 µg/ml GolgiPlug (BD PharMingen) in complete culture medium for 4 additional hours. Cells were first membrane-stained with anti-CD4. Then, cells were fixed with PBS 2% paraformaldehyde for 10 min at room temperature and intracellularly stained with conjugated-antibodies (anti-IL22, anti-IFNg, anti-IL-17A) in PBS 0.5% saponin for 45 min.

Cells were analyzed in a LSRFortessa Flow Cytometer and the data were processed with FlowJo v10.0.4 (Tree Star). Gating strategy is shown in Supplemental Fig. 1.

### RNA isolation and retrotranscription

RNA was extracted from 200 µl of plasma using miRNeasy Serum/Plasma Advanced Kit (Qiagen), following the manufacturer’s instructions. RNA was purified using RNeasy UCP MinElute spin columns, eluting with 20 µl of Rnase-free water. RNA samples were stored at − 80 °C until. Reverse transcription was performed from 2 µl of cDNA in a final reaction volume of 20 µl using miRCURY LNA RT Kit (Qiagen) according to manufacturer’s instructions. cDNA samples were stored at − 20 °C.

### RT-PCR assays and miRNA expression analysis

RT-PCR assays were performed using ready-to-use miRCURY LNA miRNA serum/plasma Focus PCR Panels and miRCURY LNA SYBR Green PCR Kit (Qiagen) attending to manufacturer’s instructions. Briefly, a mix containing 980 µl of Rnase-Free Water, 1 ml of 2 × miRCURY LNA SYBR Green Master Mix and 20 µl of cDNA template was prepared, and 10 µl was dispensed per well. A CFX384 PCR detection system (Bio-Rad) was used for the assays.

Data were analyzed using the global mean normalization method^12^. Briefly, after exclusion of values above 36, Cq values were converted to relative quantities (RQ) and sample specific normalization factor (NF) was calculated as the geometric mean of the RQs of all expressed targets per sample. Normalized Relative Quantities (NRQ) were obtained by dividing the RQs by the sample specific NF. Data were expressed as NRQ.

### miRNA target identification

miRTarBase database was used for the unravel of miRNA targets and only those under type support “Functional miRNA–target interactions (MTI)” were selected to be subjected to the PANTHER Classification System, targets with a weak functional support were excluded. A statistical test of overrepresentation for Gene Ontology biological process was performed using the complete Homo sapiens genome as reference list. Data were then analyzed by Fisher’s test and Bonferroni correction.

### Statistical analysis

Continuous variables are presented as mean ± standard deviation and compared either with the Student’s t test, ANOVA or Mann–Whitney U test depending on number of groups and the variable distribution. Normal distribution of all variables was assessed by Kolmogorov–Smirnov test. Categorical variables are expressed as absolute number and percentage and were compared with the Fisher’s exact test. Probability values of < 0.05 were considered statistically significant.

Assuming that the number of microRNAs differentially expressed among groups is very small, we ranked the miRNAs according to the fold change (high pollution/low pollution). Arbitrary we used a cut-off ≥ 1.5-fold change to select the microRNAs to be analyzed throughout the study. Differences between groups were then analyzed using Mann–Whitney U test or Kruskal–Wallis test depending on the number of groups.

In patients presenting with MI (STEMI or NSTEMI), the correlation among air pollutants was assessed by the Spearman test. Results were shown as a correlation matrix. Furthermore, in all patients, the correlation of air pollutants with immune cells was assessed by Spearman test.

### Ethical approval

This study design complied with the recommendations of the Helsinki declaration for investigation with human subjects and was approved by the Ethics Committee of La Princesa University Hospital, Madrid.

### Informed consent

All patients provided informed consent.

## Results

### Characteristic of the recruited population

A total of 249 consecutive patients (139 STEMI, 53 NSTEMI and 57 stable angina) were included. Characteristics of the population are summarized in Table 1. Compared with the other groups, patients presenting with STEMI were younger and the inclusion episode was the debut of CAD. Hypertension, dyslipidemia and diabetes were more common in the stable angina group, while active smoking was more frequent in STEMI group. Multivessel disease was more prevalent in NSTEMI group. Otherwise, no differences were observed among the groups in terms of PM_2.5_ short-term exposure.

**Table 1:** Baseline characteristics.

	STEMI (139)	NSTEMI (53)	Stable Angina (57)	p
Age (years)	62 ± 14	65 ± 12	67 ± 9	0.02
Male	109 (78)	40 (75)	57 (70)	0.47
BMI (kg/m^2^)	27.4 ± 4.2	27.6 ± 4.6	28.1 ± 5.4	0.65
Hypertension	73 (53)	33 (62)	39 (68)	0.10
Dyslipidemia	74 (53)	36 (68)	50 (88)	0.001
Diabetes mellitus	28 (20)	15 (28)	26 (46)	0.002
**Smoker**				0.001
Active	80 (57)	10 (19)	11 (19)	
Previous	26 (19)	19 (36)	26 (46)	
No	33 (24)	24 (45)	20 (35)	
Early familial coronary artery disease	13 (9)	1 (2)	2 (4)	0.12
Peripheral arterial disease	6 (4)	3 (6)	9 (16)	0.27
Previous myocardial infarction	8 (6)	10 (19)	19 (33)	0.001
Previous percutaneous coronary intervention	8 (6)	8 (15)	18 (32)	0.001
Previous coronary artery bypass graft	0 (0)	2 (4)	6 (11)	0.001
Chronic kidney disease (stage > = 3; GFR < 30 ml/min/kg)	9 (7)	3 (6)	2 (4)	0.76
**Killip-Kimball class at presentation**				
I	124 (89)	49 (93)	NA	0.70
II	8(6)	2 (4)	NA	
III	1 (1)	1 (2)	NA	
IV	6 (4)	1 (2)	NA	
**Number of diseased vessels**				0.001
0	0	3 (6)	19 (33)	
1	80 (58)	30 (57)	18 (32)	
2	38 (27)	9 (17)	11 (14)	
3	21 (15)	11 (21)	1 (2)	
Troponin T peak (ng/ml)	3900 ± 3117	1168 ± 239	NA	0.001
Creatin kinase peak (U/L)	1637 ± 1434	289 ± 221	NA	0.001
PM_2.5_ (µg/m^3^)	10.4 ± 5.8	11.9 ± 6.1	11.6 ± 5.4	0.21

Quantitative variables are shown as mean ± SD and qualitative variables are shown as frequency (percentage).

### Air pollutants

**Figure 1:**
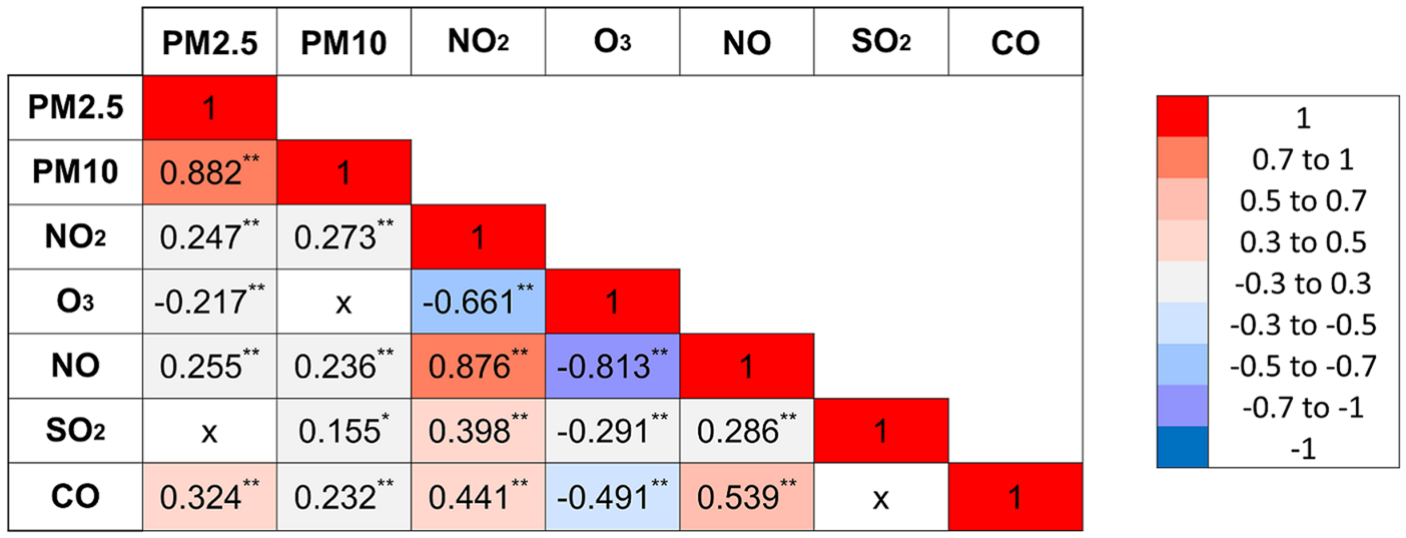
Correlation map of air pollutants. **Correlation is significant at the 0.01 level. *Correlation is significant at the 0.05 level. *X* no significant correlation.

The median (interquartile range) for each air pollutant were the following: 16.1 µg/m^3^ (13 µg/m^3^) for PM_10_, 9.5 µg/m^3^ (6.7 µg/m^3^) for PM_2.5_, 45.3 µg/m^3^ (23.2 µg/m^3^) for NO_2_, 4 µg/m^3^ (3.9 µg/m^3^) for SO_2_, 12.9 µg/m^3^ (16 µg/m^3^) for NO, 0.38 mg/m^3^ (0.14 mg/m^3^) for CO, and 51 µg/m^3^ (26 µg/m^3^) for O_3_. The correlation among the air pollutants is shown in Fig. 1. NO and NO_2_, both precursors of O_3_, had a negative correlation with O_3_. PM_2.5_, a component of PM_10_, had a positive correlation with PM_10_. PM_2.5_ and NO_2_ had a low positive correlation.

### Circulating inflammatory cell analysis

**Figure 2:**
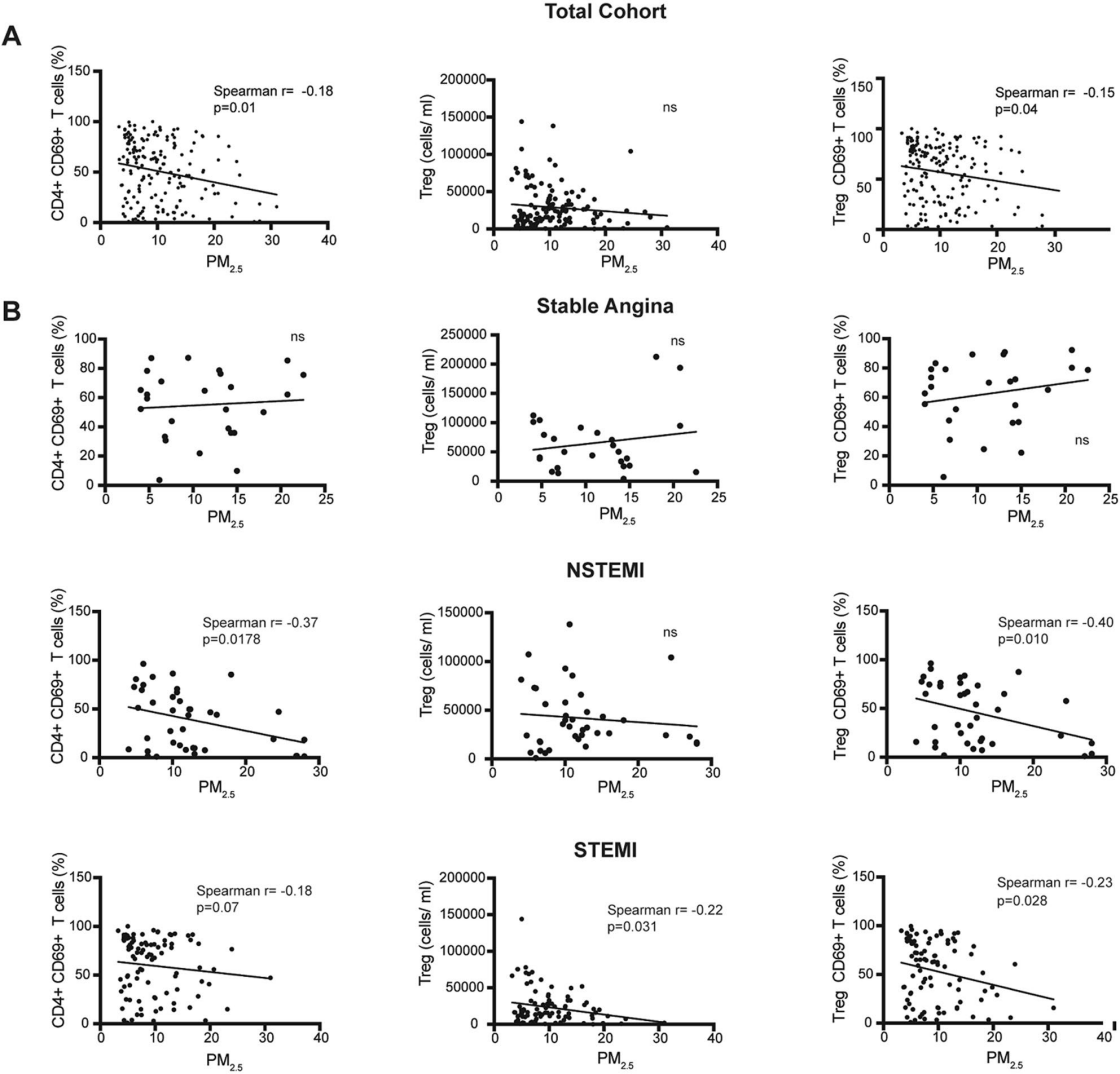
Correlation of PM_2.5_ with CD69 + CD4 T cells and Treg cells. Scatter plots are shown. (**A**) Association of PM_2.5_ exposure with CD4 + T cell subsets in the whole cohort. (**B**) Association of PM_2.5_ exposure with CD4 + T cell subsets in stable angina, NSTEMI and STEMI patients. Correlation was assessed by Spearman test.

To determine the association of air pollutant exposure and the immune response, we performed correlation analysis with different subsets of T lymphocytes in the whole cohort. No association between total CD4 + T cells and PM_2.5_ were detected. However, a negative correlation between CD4 + CD69 + T cells and PM_2.5_ exposure was observed (r = − 0.18, p = 0.01) (Fig. 2A). Interestingly, although PM_2.5_ in the whole cohort was not associated with total number of T cells, we observed a negative association with the percentage of Treg CD69 + T cells (r = − 0.15, p = 0.04) (Fig. 2A). We wonder whether these air pollution-associated changes were occurring in the different clinical presentation of atherosclerosis disease. Remarkably, the reduction of both CD4 + CD69 + and Treg CD69 + T cells was observed in NSTEMI and STEMI patients but not in stable angina (Fig. 2B). Regarding T cells producers of IL-22 and IL-17 no association was detected with PM_2.5_ in the whole cohort or the different clinical presentations.

**Figure 3:**
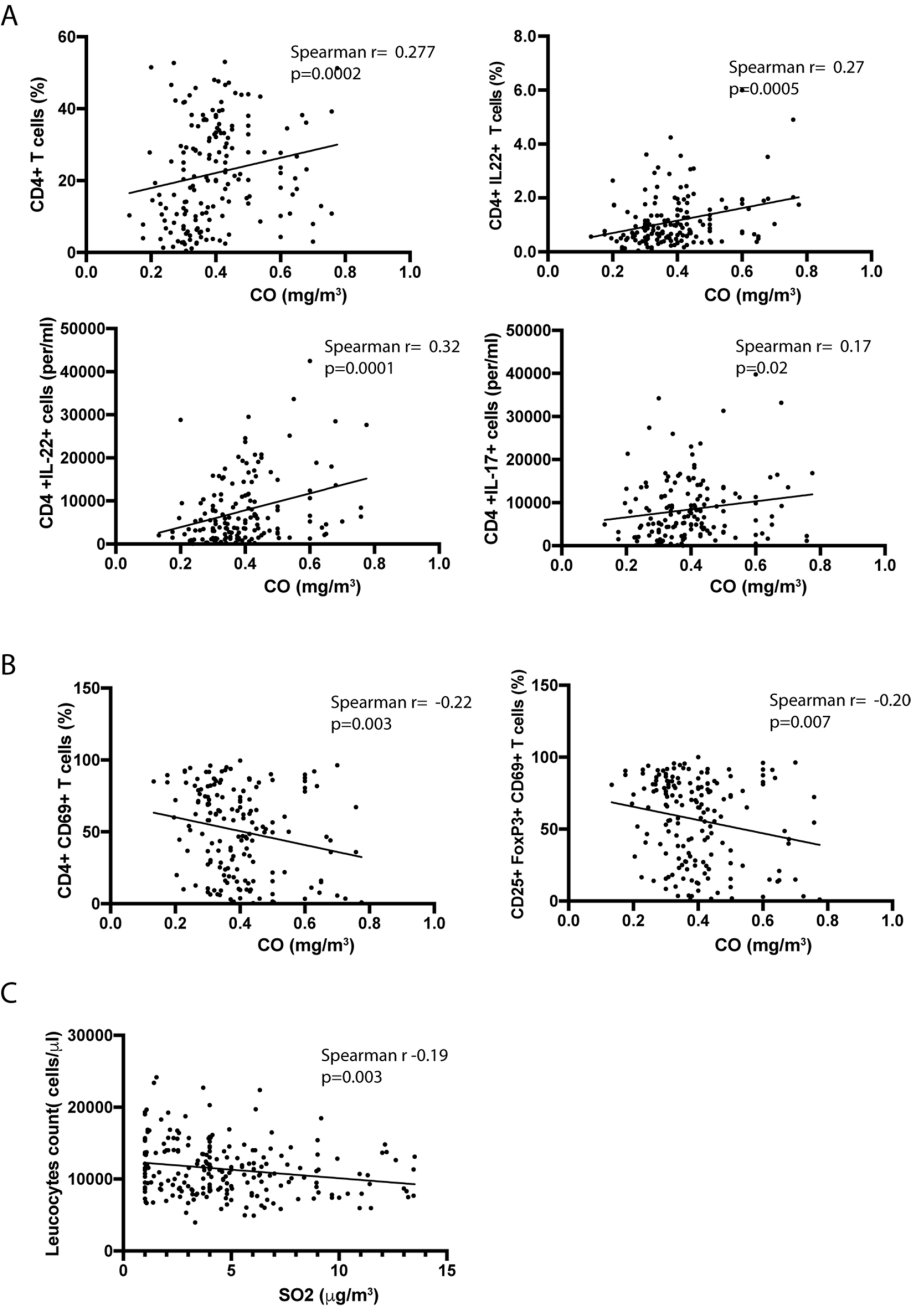
Correlations of CO and SO_2_ exposure with circulatory immune cells. Scatter plots of significant correlations are shown. Correlation was assessed by Spearman test. (**A**) CO exposure had a positive correlation with the percentage of CD4^+^T cells as well as with IL22- and IL-17-producers CD4^+^ T cells. (**B**) Negative correlation of CO exposure with CD4^+^CD69^+^ lymphocytes and CD69^+^ Treg cells. (**C**) SO_2_ exposure was associated with a decrease of total leucocytes count.

In addition, associations of NO, NO_2_, O_3_, CO and SO_2_ levels with the immune response was explored. High levels of CO were associated with an increase of the percentage of peripheral blood CD4 + T cells (r = 0.27, p = 0.0002) and, specifically, with the percentage of T cells producers of IL-22 (r = 0.27, p = 0.0005) (Fig. 3A). Moreover, exposure to high levels of CO was also associated with a high number of CD4^+^IL-22^+^ cells per ml of blood (r = 0.32, p = 0.0001) (Fig. 3A). A weaker but significant correlation of CO exposure with the numbers of CD4^+^IL-17^+^ cells per ml of blood was observed (r = 0.17, p = 0.02 Fig. 3A). On the contrary, the expression of the anti-inflammatory CD69 receptor on total CD4^+^ T cells and CD4^+^CD25^+^Foxp3^+^ regulatory T cells (Treg) showed a negative correlation with CO exposure (r = − 0.22, p.003 and r = − 0.20, p = 0.007 respectively) (Fig. 3B). Regarding the SO_2_, our data showed a weak but significant negative association with the total count of peripheral blood leucocytes (r = − 0.19, p = 0.003, Fig. 3C). Nevertheless, no significant associations were detected between the analyzed populations and the rest of air pollutants evaluated (NO, NO_2_ and O_3_).

### miRNA analysis

A total of 31 patients were selected for this analysis, 17 of them exposed to low pollution and 14 exposed to high pollution. Eight out of 31 patients had diagnosis of stable angina, 9 NSTEMI and 14 STEMI. Assuming that the number of microRNAs differentially expressed among different groups would be small, we first calculated the fold change of miRNA expression between high and low pollution. We identify 22 microRNAs with a fold change ≥ 1.5 However, only 9 out of 22 microRNAs showed statistically significant differences between patients exposed to high levels of pollution compared to the exposure to low levels (Table 2).

**Table 2:** Top microRNAs expressed in patients exposed to high levels of pollution.

miRNA ID	Fold change*	p value**
hsa-mir-127-3p	5.44	0.185
hsa-mir-409-3p	3.94	**0.016**
hsa-mir-136-5p	3.46	0.124
hsa-mir-376c-3p	2.78	**0.003**
hsa-mir-382-5p	2.66	**0.030**
hsa-mir-485-3p	2.32	0.605
hsa-mir-376a-3p	2.28	**0.023**
hsa-mir-136-3p	1.97	0.298
hsa-mir-495-3p	1.90	0.090
hsa-let-7f-5p	1.82	**0.002**
hsa-mir-106b-3p	1.81	0.090
hsa-mir-199a-5p	1.76	0.099
hsa-mir-28-5p	1.76	0.074
hsa-mir-146a-5p	1.65	0.164
hsa-mir-501-3p	1.64	**0.027**
hsa-mir-423-3p	1.61	**0.029**
hsa-mir-766-3p	1.60	0.255
hsa-mir-1	1.59	0.069
hsa-mir-543	1.54	0.161
hsa-mir-199a-3p	1.52	0.056
hsa-mir-328-3p	1.51	**0.033**
hsa-mir-335-5p	1.47	**0.026**

*Exposure to high pollution/low pollution, **Mann–Whitney U t-test, bold indicate p < 0.05.

Interestingly, the functional profiling of the microRNA gene targets listed in Table 2 showed an enrichment mainly in biological processes associated with development and morphogenesis of cardiovascular system as well as in several processes of inflammatory response (Table 3). Indeed, more than 50% of the top 50 biological processes enriched in genes regulated by our list of miRNAs correspond to the cardiovascular system biology and immune response pathways.

**Table 3:** Top 25 over-represented functional classes for miRNA targets.

Gene ontology biological process	p value *
Neg regulation of cardiac muscle cell differentiation	3.51E−04
Neg regulation of cardiocyte differentiation	1.04E−04
Pos regulation of cardiac epithelial to mesenchymal transition	1.53E−02
Coronary artery morphogenesis	1.53E−02
Foregut morphogenesis	2.35E−02
Regulation of cardiac epithelial to mesenchymal transition	2.35E−02
Regulation of Wnt signaling pathway involved in heart development	2.35E−02
Negative regulation of cell size	3.47E−02
Positive regulation of fibroblast migration	3.63E−04
Notch signaling involved in heart development	4.98E−02
Regulation of cardiac muscle cell differentiation	7.42E−04
Positive regulation of macrophage differentiation	7.16E−03
Positive regulation of ER unfolded protein response	7.16E−03
Regulation of cardiocyte differentiation	2.44E−07
Pericardium development	1.49E−04
Negative regulation of DNA damage response	9.88E−03
Pos regulation of leukocyte adhesion to vascular endothelial cell	1.49E−04
Regulation of cell communication by electrical coupling	9.88E−03
Positive regulation of nitric-oxide synthase biosynthetic process	1.34E−02
Cell surface receptor sig pathway involved in heart development	2.73E−04
Aortic valve morphogenesis	8.17E−07
Regulation of nitric-oxide synthase biosynthetic process	2.53E−03
Positive regulation in cellular response to chemical stimulus	3.62E−04
Regulation of leukocyte adhesion to vascular endothelial cell	6.83E−05
Interleukin-6-mediated signaling pathway	2,35E−02

*Bonferroni adjusted p value.

**Figure 4:**
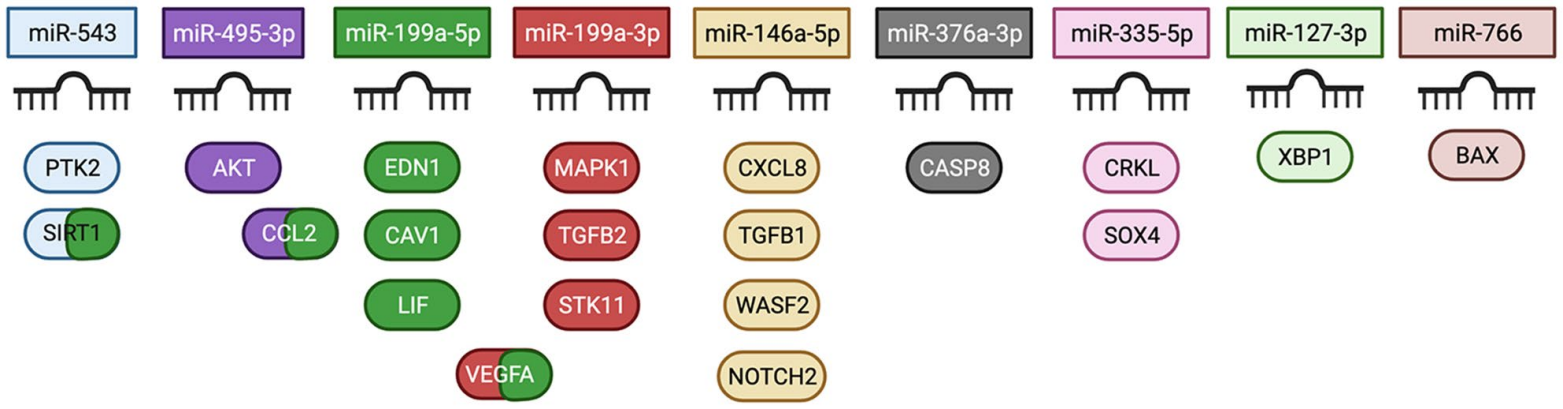
Target genes of differentially expressed genes in CAD patients exposed to high levels of PM_2.5_. Functional miRNA targets associated to both Cardiovascular System and Immune System are shown. Different colors are used to indicate the targets that are regulated by each miRNA. From all genes identified in miRTarBase as targets of microRNAs listed in Table 2, only those with functioonal support were selected to perform the enrichment analysis. Image created with BioRender.com.

Remarkably, nine out of 22 top overexpressed microRNAs regulate the expression of genes involved in both biological processes (Fig. 4).

**Figure 5:**
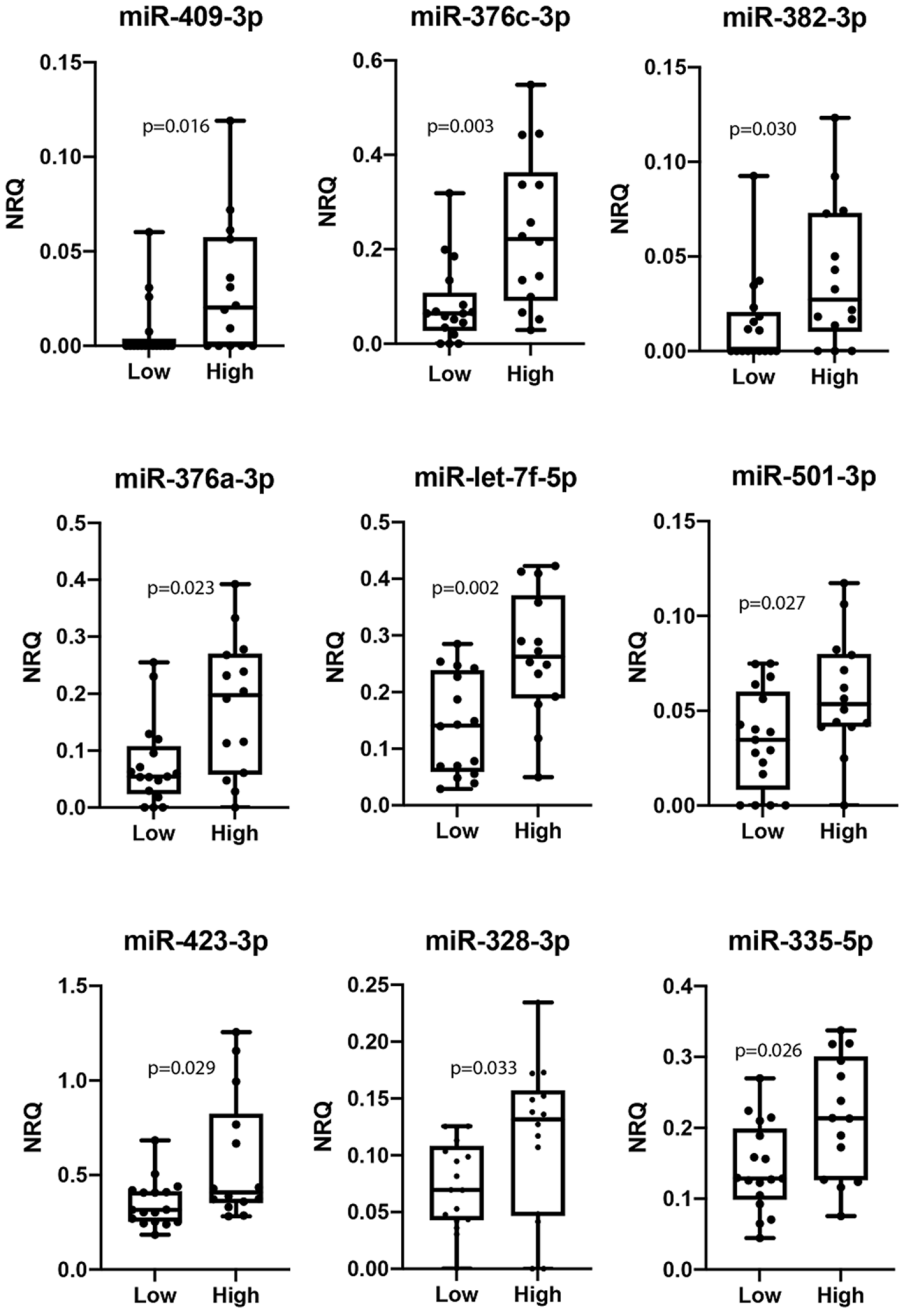
Differential expression of miRNAs in plasma samples from CAD patients exposed to low and high levels of PM_2.5_. Box and whiskers Min to Max plots showing plasma levels of microRNAs from CAD patients (n = 31) exposed to low or high levels of PM_2.5_. Including all clinical presentations in the analysis, high PM_2.5_ short-term exposure was associated with (**A**) increased and (**B**) decreased miRNA expression. Differences were analyzed using Mann–Whitney U test.

Higher circulating levels of miR-409-3p, miR-376c-3p, miR-382-5p, miR-376a-3p, miR-let-7f-5p, miR-501-3p, miR-423-3p, miR-328-3p and miR-335-5p (all with a p value < 0.05) were detected in those patients exposed to high levels of pollution (Fig. 5).

**Figure 6:**
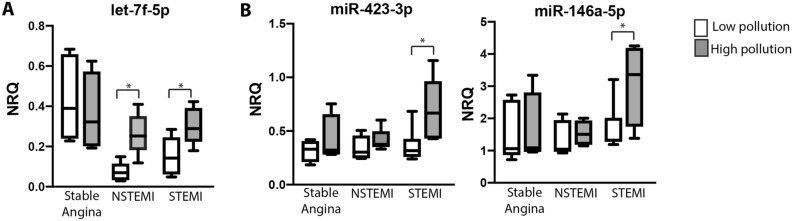
Circulating levels of miR-let-7f-5p, miR-423-3p and miR-146a-5p are increased in acute myocardial patients exposed to high levels of PM_2.5_. Box and whiskers Min to Max plots showing the expression of (**A**) miR-let-7f-5p and (**B**) miR-423-3p and miR-146a-5p in plasma samples from stable angina patients (n = 8), NSTEMI patients (n = 9) and STEMI patients (n = 14) exposed to low levels (empty boxes) or high levels (grey boxes) of PM_2.5_. Differences were analyzed using Mann–Whitney U test, *p < 0.01.

Subsequently, we analyzed the changes in circulating miRNAs associated to pollution in each clinical presentation. Interestingly, we observed that expression of miR-let-7f-5p was increased in NSTEMI and STEMI patients exposed to high levels of pollution, while no significant changes were detected in patients with stable angina (Fig. 6A). In addition, exposure to high pollution was significant associated with higher levels of miR-423-3p and miR-146a-5p only in STEMI patients (Fig. 6B).

## Discussion

This is the first study systematically assessing the biological changes in peripheral blood CD4 + T cell and circulating miRNAs, associated with short term exposure to air pollutants in patients with MI. CAD characterizing our population represents a relevant difference with the previous reports, since in most studies healthy participants had been recruited to assess the biological response to air pollutants^11^. Nevertheless, healthy participants and patients with CAD may have a different response to exogenous stressors. Besides, previous studies exploring biomarkers in MI patients do not usually include pollution as a variable^13^.

Plaque destabilization may lead to a wide range of clinical presentations, from asymptomatic plaque rupture or erosion to occlusive atherothrombosis. Thrombogenicity, inflammation, oxidative stress and endothelial function have a large variability in response to exogenous and endogenous stimulus leading to a multifaceted vulnerability milieu that eventually explains the final clinical presentation resulting from acute plaque destabilization^14^. Notably, air pollution appears to participate in all the stages of this vulnerable state^8^.

In the circulating white cell analysis, CO was associated both with an increased number of CD4 + cells producers of IL-17 and IL-22. These findings are consistent with a previous study which analyzed white blood cell changes in patients with chronic respiratory disease exposed to CO in the previous 24 h, observing increased lymphocytes counts^15^. Interestingly, IL-22 and IL-17 expression are induced by the activation of aryl hydrocarbon receptors, a transcription factor that is a target for pollution^16^. High SO_2_ short-term exposure was associated with reduction in the total leucocytes count. In a previous animal model using inhaled SO_2_, this finding was also reported^17^. Remarkably, both PM_2.5_ and CO short-term exposures were associated to CD69^+^Treg cells reduction. In this regard it is important to highlight that the immunosuppressive activity of Treg cells is increased in those cells expressing CD69^18^. Moreover, PM_2.5_ exposure in STEMI patients was associated with a reduction in Treg cells. These findings are relevant as numerous studies showed that Tregs deficiency or dysfunction are associated with the development of atherosclerosis^19,20^ that may be related to the protective effect of Tregs on PM-induced inflammatory response^21^. There is no previous data about the effects of air pollution on Treg cell in patients with CAD. Nevertheless, a similar decreased expression of FOXP3 has been described in atopic children exposed to air pollutants^22^. Recently, our group identified the protective role of CD69 for atherosclerotic disease, and peripheral leucocytes from subclinical atherosclerosis individuals express low level of this molecule^23^. In this regard, our current data strongly suggest that exposure to air pollutants is associated with a reduction in CD69 in T cells.

In the miRNA analysis, we found several miRNA altered by PM_2.5_ short-term exposure. Interestingly, all of them were linked to gene expression involved in cardiovascular or immune system processes participating in the atherosclerotic disease. Remarkably, few of them were specifically modified only in patients presenting with an acute MI: miR-let-7f-5p was increased in patients with STEMI or NSTEMI, while miR-423-3p and miR-146a-5p were only increased in the STEMI group.

The let-7 family is highly expressed in the cardiovascular system, being miR-let-7f related to angiogenesis, ischemia, arrhythmia and heart development^24^. Recently, upregulation of miR-let-7f-5p has been documented in activated platelets^25^. In a previous report, stress cardiomyopathy showed higher levels of miR-let-7f-5p compared to STEMI, arguing that the observed difference, among others, may be related to alteration of the microcirculation^13^. However, no data of pollutant exposure have been reported.

In a large Chinese cohort of general population in primary prevention, lower levels of circulating miRNA-423-3p predicted acute MI in the follow up, performing better than hs-CRP. Unfortunately, the study was missing pollution data. In addition, an in vitro research in rat cardiac fibroblasts documented a possible involvement of miRNA-423-3p in the ischemia–reperfusion injury^26^.

The observed upregulation of miR-146a was previously reported in steelworkers after short term exposure to PM_2.5_^27^. In addition, miR-146a-5p was previously proposed as a biomarker of PM-induced impaired inflammatory response^28^. miR-146a is a cytokine-responsive miRNA induced by TNF-α and interleukin-1β. In experimental atherosclerosis its overexpression inhibits cytokine responsiveness of endothelium, suggesting that it could be part of a negative feedback mechanism limiting endothelial cell inflammatory signaling^29^. Remarkably, miR-146a is a crucial regulator of T reg suppressive function preventing the conversion of Tregs in IFNγ-producing Th1-like cells^30^. In addition, miRNA-146a regulates the maturation process and pro-inflammatory cytokine secretion by targeting CD40L in oxLDL-stimulated dendritic cells^31^. Despite the miR-146a upregulation, its anti-inflammatory effect may be ineffective in polluted areas as PM_2.5_ short-term exposure can silence genes by DNA methylation of CpG islands of promoters^32,33^. At any rate, the concomitant Tregs reduction and miR-146a increase observed in STEMI patients with short-term exposure to PM_2.5_ seems to be strictly related and may represent a characterizing pattern of pollution-associated STEMI.

Altogether, our results strongly suggest a modulating effect of short-term exposure to air pollutants on circulating immune cells and miRNA expression in patients with CAD. These changes may participate in the increased risk of STEMI and worse outcomes in people exposed to air pollutants.

Our study has several limitations that should be acknowledged. Despite the significant differences, due to the small sample size, our study should only be considered as hypothesis generating and results should be confirmed in larger studies. Moreover, a selection bias cannot be excluded. In addition, confounder effects of lipids, diabetes status, smoking, nutrition, social status and cardiovascular drugs use, could not be rule out.

Furthermore, PM_2.5_ components may vary significantly depending on the different sources of pollution and the specific climate conditions of the geographical area^34^. Madrid is the most populous city in Spain (6 million people in the urban area) with a climate of transition between the Mediterranean and the cold semi-arid climate. Like other capitals in developed countries, its main sources of air pollution are motorized road traffic, apart from commercial and residential heating. Therefore, our results could not be extrapolated to areas with different characteristics.

In addition, polymorphisms, haplotypes and variability in plasma levels of C-reactive protein, fibrinogen, IL-6 may have altered the pro-inflammatory response of air pollution in myocardial infarction patients^35^. Finally, because their role in atherosclerosis, innate immune cells should also be explored in further studies. Furthermore, since all participants had an established coronary artery disease, the observed inflammatory changes in response to air pollutants cannot be generalized to healthy individuals. Despite these limitations, our study provides novel unique insights on the mechanisms involved in the pathogenesis of acute MI associated with short-term exposure to air pollutant. Further studies are warranted for a more complete understanding of the physiopathology of this process in order to inform clinical decisions and develop prevention strategies aimed to reduce the risk of MI in patients exposed to air pollution.

In conclusion, our study identifies circulating inflammatory cells and the miRNA changes of acute MI related to short-term exposure to air pollutants. Specifically, STEMI related to PM_2.5_ short-term exposure is associated to specific changes involving CD4^+^CD25^+^Foxp3^+^ Treg cells and miR-146a-5p.

## Supplementary Information

Supplementary Information 1.

Supplementary Information 2.

## Data Availability

Data sharing will be considered upon reasonable request including a detailed research plan with the corresponding author.
