# Supplementary figures and images for: Influence of air pollutants on circulating inflammatory cells and microRNA expression in acute myocardial infarction
Source: Sci Rep. 2022 Mar 30;12:5350. doi: 10.1038/s41598-022-09383-7 (PMC8967857; doi:10.1038/s41598-022-09383-7)

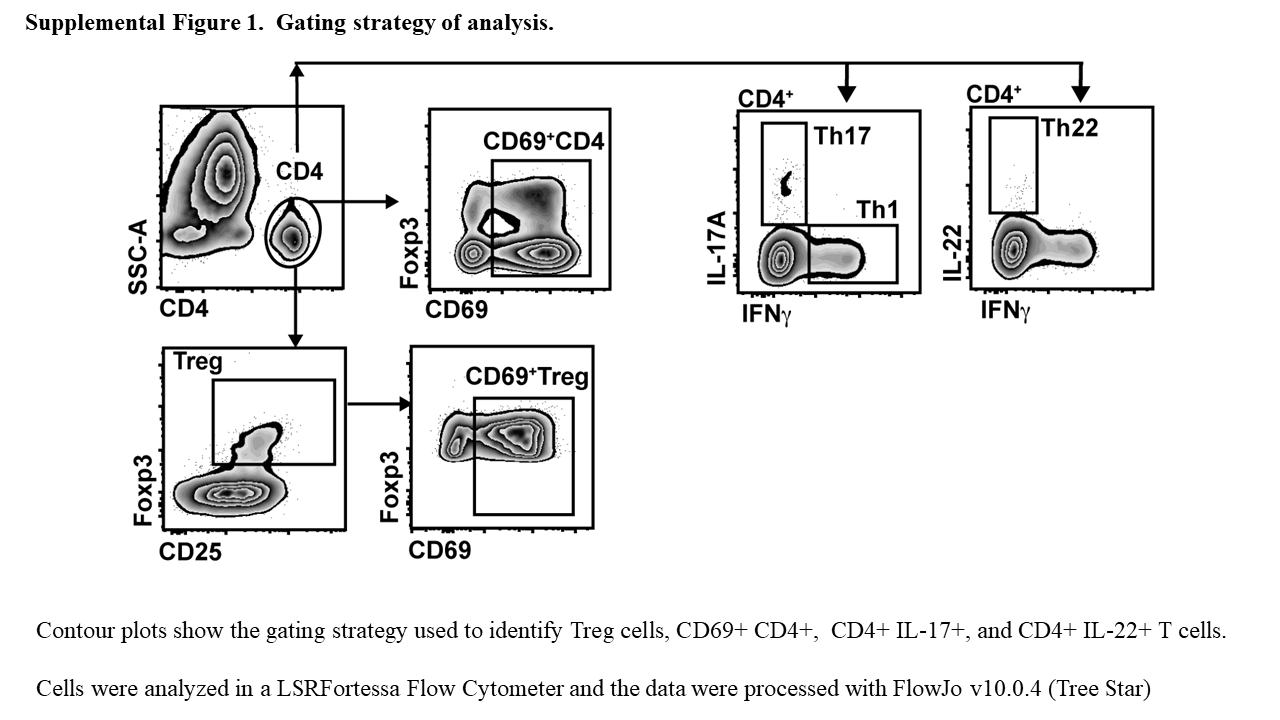

Supplement: Supplementary file 1 — Supplementary Information 1. [file 41598_2022_9383_MOESM1_ESM.tif]
