# Supplementary material for: Influence of air pollutants on circulating inflammatory cells and microRNA expression in acute myocardial infarction
Source: Sci Rep. 2022 Mar 30;12:5350. doi: 10.1038/s41598-022-09383-7 (PMC8967857; doi:10.1038/s41598-022-09383-7)
Supplement: Supplementary file 2 — Supplementary Information 2. [file 41598_2022_9383_MOESM2_ESM.docx]

**Supplemental Table 1. List of human fluorochrome-conjugated antibodies for flow cytometry analysis used in this study.**

| **Marker** | **Clone** | **Manufacturer** |
| --- | --- | --- |
| Human CD25-APC | 2A3 | BD Biosciences |
| Human CD4-PE-Cy7 | SK3 | BD Biosciences |
| Human CD66b-APC | G10F5 | Biolegend |
| Human CD69-V421 | FN50 | BD Biosciences |
| Human Foxp3-PE | 3G3 | Miltenyi Biotec |
| Human HLA-DR-PE | G46-6 | BD Biosciences |
| Human IFNγ-FITC | B27 | BD Biosciences |
| Human IL-17A-APC | SCPL1362 | BD Biosciences |
| Human IL-22-PE | 142928 | R&D Systems |
